# Identification and validation of potential prognostic biomarkers in glioblastoma via the mesenchymal stem cell infiltration level

**DOI:** 10.3389/fonc.2024.1406186

**Published:** 2024-09-02

**Authors:** Shengyu Wang, Senlin Mao, Xiaofu Li, Dan Yang, Yinglian Zhou, Hui Yue, Bing Li, Wei Li, Chengyun Li, Xuemei Zhang

**Affiliations:** ^1^ Department of Neurology, The Second Affiliated Hospital of Harbin Medical University, Harbin, China; ^2^ Department of Magnetic Resonance Imaging, The Second Affiliated Hospital of Harbin Medical University, Harbin, China; ^3^ Department of Neurology, Heilongjiang Hospital, Harbin, China

**Keywords:** glioblastoma, mesenchymal stem cell, tumor microenvironment, immune checkpoint, prognostic model

## Abstract

**Aims:**

Mesenchymal stem cells (MSCs) are key components in promoting glioblastoma (GBM) progression. This study aimed to explore new therapeutic targets and related pathogenic mechanisms based on different MSCs infiltration levels in GBM patients.

**Methods:**

We estimated the relationship between cell infiltration and prognosis of GBM. Subsequently, key risk genes were identified and prognostic models were constructed by LASSO-Cox analysis. The risk genes were validated by five independent external cohorts, single-cell RNA analysis, and immunohistochemistry of human GBM tissues. TIDE analysis predicted responsiveness to immune checkpoint inhibitors in different risk groups.

**Results:**

The MSCs infiltration level was negatively associated with survival in GBM patients. LOXL1, LOXL4, and GUCA1A are key risk genes that promote GBM progression and may act through complex intercellular communication.

**Conclusion:**

This research has provided a comprehensive study for exploring the MSCs infiltration environment on GBM progression, which could shed light on novel biomarkers and mechanisms involved in GBM progression.

## Introduction

Glioblastoma (GBM) is one of the most commonly reported malignancies worldwide, and the need to improve its prognosis remains a major clinical challenge. GBM accounts for 48.6% of central nervous system malignant tumours, with a median overall survival (OS) time of 15 months (1). The tumor growth and progression effect of mesenchymal stem cells (MSCs) in GBM was demonstrated in previous studies (2, 3). However, there is a lack of analyses based on gene expression at different infiltration levels of MSCs.

MSCs have been found to migrate toward tumours, interact with the TME and promote tumor growth (2). MSCs are induced to differentiate into pericytes and promote angiogenesis in GBM. They also shift glioma stem cells (GSCs) toward a more aggressive status (4). Hossain et al. showed that MSCs isolated from fresh human brain GBM tissue can promote the proliferation and self-renewal of GSCs, thus driving the construction of an environment conducive to tumor growth. In the GBM microenvironment, about 10% of MSCs may be differentiated from GSCs (3). As a result of β-connexin phosphorylation and Wnt signalling activation in tumours, endothelial cells acquire the ability to transform into MSC-like cells and induce tumour resistance to cytotoxic treatments (2, 5).

GBM is a “cold tumor” with a tumor immune phenotype that is characterised by poor function and severe exhaustion of T cells in the TME (7–9). Considering that many immune cells and stromal cells in the TME interact to promote tumor development, we wondered whether these factors are related to cold tumours. Previous studies have observed poor T-cell function and severe exhaustion in GBM, which is characterised by upregulation of multiple immune checkpoints, T-cell hypo-responsiveness, and a low infiltration state of T cells (10). Immune checkpoint inhibitors (ICIs) promote the antitumor immune response by inducing suppressive immune checkpoint regulatory pathways and have been applied to many kinds of cancer types in the clinic (11). PD-1 blocker therapy adjuvantly enhanced local and systemic anti-tumour immune responses in glioblastoma patients, significantly increasing their overall survival (12). A study indicated that PD-L1 blockade combined with a DC vaccine can increase antitumor efficacy in a mouse model of glioma (13).

Although these tumor-promoting effects of MSCs have been observed consistently, the underlying changes in gene expression are still unknown. There is also a lack of typing studies based on MSC infiltration level indicators in GBM. This study will help identify genes that play a key role in promoting tumor progression during GBM development, screen for new therapeutic targets, and provide new ideas for the risk stratification and treatment of GBM patients.

## Materials and methods

### Data accessing and preprocessing

The RNA-seq data and clinical details of GBM patients were obtained from the TCGA database, GEO database and CGGA database. Samples without clinical information were excluded. The source and number of people in the training and validation cohorts are shown in **Table 1**. The raw counts of the training cohort (TCGA-GBM, n=158) was obtained from TCGA database. According to the requirements of the xCell website, the gene expression of the training cohort (TCGA-GBM) was normalised to transcripts per kilobase million (TPM) for xCell analysis. For subsequent differential gene analysis, the raw counts of the training cohort (TCGA-GBM) were normalised by “DESeq” function in the “DESeq2” R package (14). For validation cohort 1 (GSE74187, n=60) and validation cohort 5 (GSE16011, n=154) the data format provided by the “Series Matrix File(s)” in the GEO database was used. For validation cohort 2 (mRNAseq-325, n=137) and validation cohort 4 (mRNAseq-693, n=237), FPKM values provided by the CCGA website were used. For validation cohort 3 (mRNA-array-301, n=114), the processed format provided in the CCGA website was used.

**Table 1:** Source and number of patients in training and validation cohorts.

Group	Source	Alive n(%)	Dead n(%)	Radiation therapy n(%)	Chemotherapy therapy n(%)
Training cohort	TCGA-GBM	29(18.4%)	129(81.6%)	69(43.7%)	56(35.4%)
Validation cohort 1	GSE74187 (GEO)	14(23.3%)	46(76.7%)	Not reported	Not reported
Validation cohort 2	mRNAseq-325(CGGA)	13(9.4%)	124(90.5%)	100(73.0%)	99(72.3%)
Validation cohort 3	mRNA-array-301(CGGA)	17(14.9%)	97(85.1%)	97(85.1%)	61(53.5%)
Validation cohort 4	mRNAseq-693 (CGGA)	27(11.4%)	210(88.6%)	193(81.4%)	99(84.0%)
Validation cohort 5	GSE16011 (GEO)	7(4.5%)	147(95.5%)	119(77.3%)	10(6.4%)

The **Table 1** shows the data sources and specific details of this study, including dataset number, survival status, radiotherapy and chemotherapy treatment.

### Immune infiltration assessment and prognostic grouping

The training cohort (TCGA-GBM) was analysed by the xCell method (15). The patients were grouped according to each cell type in xCell and divided into two groups (high and low) according to the median infiltration score. Then, we evaluated the relationship between each cell type and prognosis through Kaplan–Meier (K-M) survival analysis and univariate Cox regression analysis.

### Differentially expressed gene analysis and enrichment analysis

To investigate the differences in gene expression at different levels of MSC infiltration, we divided the training cohort into two groups according to the median MSC infiltration score and performed differential analysis by the “DESeq2” R package. The screening conditions were restricted to |fold change| > 2, p < 0.05, and adjusted p < 0.05. To explore the potential biological functions of the up-regulated differentially expressed gene (DEGs), we further conducted Kyoto Encyclopedia of Genes and Genomes (KEGG) and Gene Ontology (GO) enrichment analyses.

### Screening for the prognostic risk genes

To screen for genes strongly associated with prognosis, we performed univariate Cox regression analysis and K-M survival analysis. The least absolute shrinkage and selection operator (LASSO) analysis was employed, followed by 10 cross-validations with the “glmnet” R package.

### Establishment of the GBM prognostic model

To construct the final prognostic model and determine the independence of the genes included in the model, we performed multivariate Cox regression analysis (16). The prognostic model of GBM was as follows: Risk score=∑expgenei* βi , where expgenei is the expression of each chosen risk gene and βi is the regression coefficient. A risk score was calculated from this model for each patient. We separated the patients into two groups (high- and low-risk groups) according to the cutoff point of the risk score by “maxstat” R package.

### Single-cell RNA analysis

The single-cell sequencing data and cell markers used were taken from files in the supplementary file of the GEO database (17). The “Seurat” package was utilised to generate objects and filter out poor quality cells while performing standard data preprocessing procedures (16). Percentages of gene count, cell count and mitochondrial content were calculated. The filtering criterion was to detect cells with less than 20 detected genes. Retained genes detected in at least 1 cell. Filter out cells with less than 100 or more than 15,000 detected genes and cells with high mitochondrial content (>20%). We scaled the UMI counts using scale.factor = 10000. After logarithmic transformation of the data, the ScaleData function in “Seurat (v4.4.0)” was used. The corrected normalised data metrics were applied to the standard analysis. The first 2000 variable genes were extracted for principal component analysis (PCA). We performed cell clustering using the FindClusters function implemented in the “Seurat” R package (resolution = 2.0). “CellChat” package is an R package that can predict intercellular communication networks from single-cell RNA sequencing data (18). By inputting gene expression, signal ligands, receptors, and their co-factors, “CellChat” package can predict intercellular communication and visualise the results, which provides more meaningful information to help us understand the concrete mechanism.

### Evaluation of predictive performance

K-M survival analysis was performed to evaluate the association between GBM risk grouping and overall survival and to check the predictive capability of our model. The log-rank test was used to determine significance. The prognostic areas under the curve (AUCs) at 1, 3, and 5 years were calculated with the “timeROC” R package. The above analysis methods were applied to the training cohort and validation cohorts. We compared the expression of immune checkpoints and related ligands in all cohorts between the two groups, including PDCD1 (PD1), CTLA4, HAVCR2 (TIM-3), TIGIT, BTLA, CD274 (PD-L1), PDCD1LG2 (PD-L2), and CD80 (19), and predicted the response to ICI treatment by TIDE (http://tide.dfci.harvard.edu/). TIDE was used to assess the possibility of immune escape in tumours. Higher TIDE scores indicate an inferior treatment response to ICIs (20).

### Prediction of interaction genes and functional enrichment analysis

To explore the potential mechanism, the interaction genes of model genes were predicted by GeneMANIA (http://genemania.org/) (21). In terms of biological function, the model genes and the predicted interaction genes were analysed using the KEGG pathway analysis and the GO enrichment method.

### Haematoxylin and eosin and immunohistochemistry staining in GBM

With the human subjects’ understanding and consent and the approval of the Ethics Committee of the Second Hospital of Harbin Medical University (approval number: KY2022-001), we collected the magnetic resonance imaging (MRI) images and pathological sections of 6 GBM patients. Three of them were patients with OS < 15 months and the other three were patients with OS ≥ 15 months. Standard HE staining was performed to observe the GBM tissue structure. To observe the expression and location of the 3 risk genes, we performed IHC staining, which was conducted on GBM paraffin sections by using an anti-LOXL1 polyclonal antibody (1:400, PB0758, Boster, China), anti-LOXL4 polyclonal antibody (1:50, TW11440, Shanghaitongwei, China) or anti-GUCA1A antibody (1:300, E-AB-53078, Elabscience, China). ImageJ was used for semi-quantitative analyses and Graphpad prism for histograms.

### Statistical analysis

Plotting and statistical analysis were performed by R (4.0.5) and SPSS. The log-rank test was used to compare the significance of differences in component K-M survival analysis. The Wilcoxon test was used to compare gene expression between the two groups. A t test was used to compare TIDE scores between the two groups. A p value < 0.05 was considered significant.

## Results

### Worse survival situation in the higher MSC infiltration group

**Figure 1:**
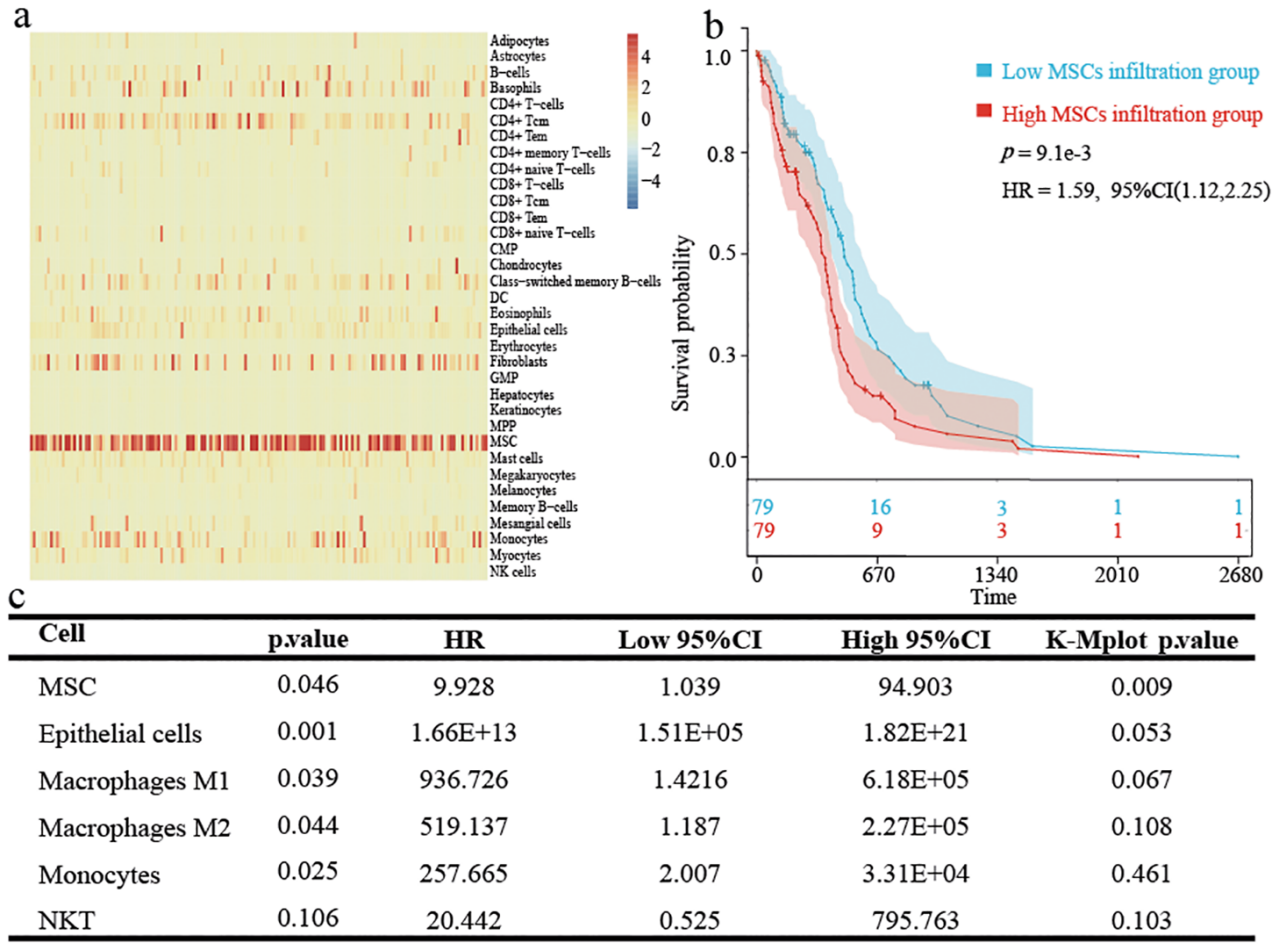
Immune cell and stromal cell infiltration in TCGA-GBM patients. **(A)** Heatmap showing xCell assessment in GBM patients. **(B)** Kaplan-Meier survival analysis based on the grouping at the median value of MSCs infiltration scores in xCell. **(C)** The results obtained by univariate cox regression analysis and K-M survival based on each cell infiltration are presented by triliniear plots.

The heatmaps clearly present the first 34 cells in the xCell assessment (**Figure 1A**). Many immune cells were infiltrated to a lesser extent, and MSCs were more significantly infiltrated than other cells. K-M survival analysis and univariate Cox regression showed that higher MSC infiltration was significantly related to worse survival (**Figures 1B, C**).

### Differential analysis and enrichment analysis

**Figure 2:**
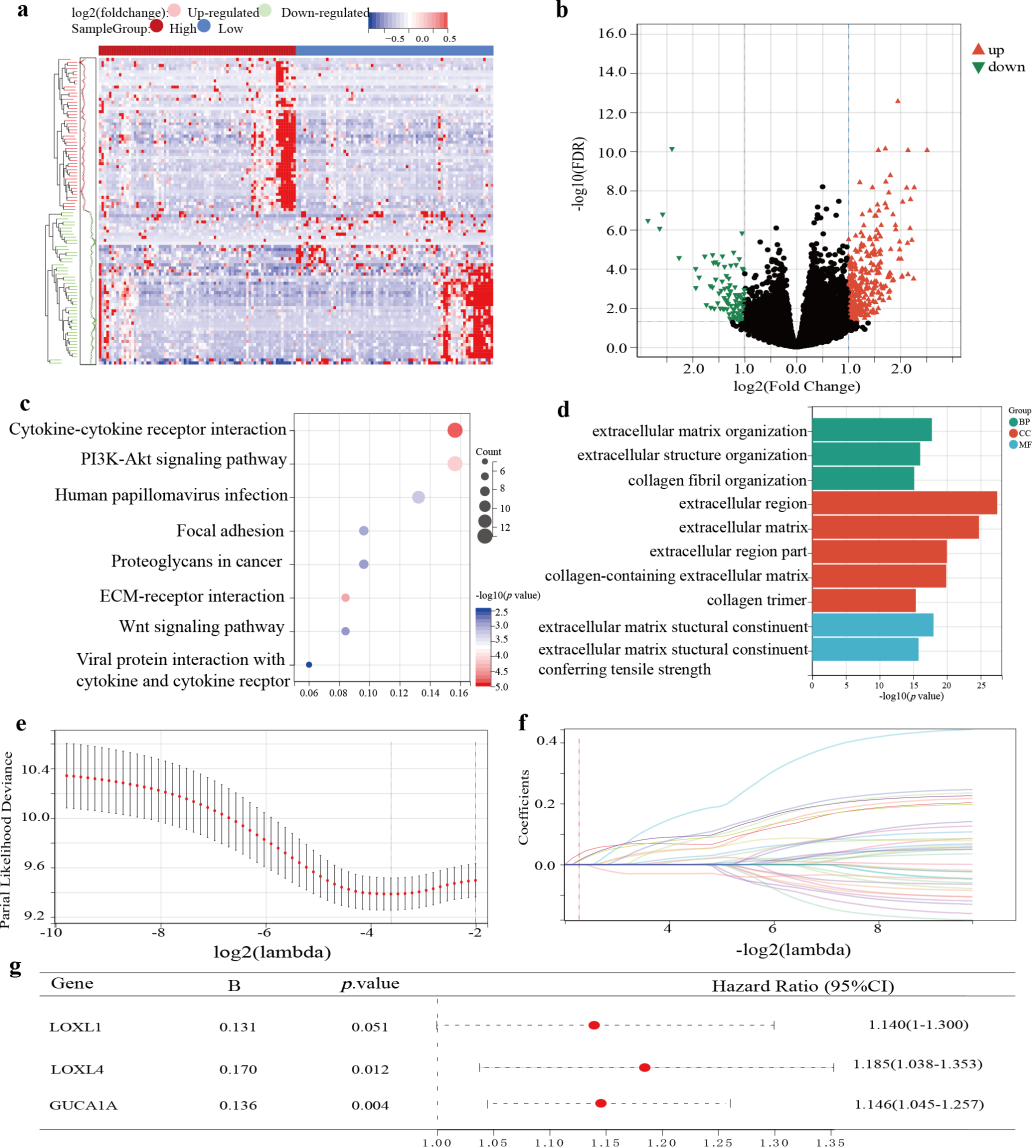
Identification and establishment of TCGA-GBM prognostic model. **(A)** The expression of DEGs based on the grouping at the median value of MSCs infiltration score is shown by heatmap. **(B)** Screening conditions for DEGs. **(C)** KEGG enrichment analysis for up-regulated DEGs. **(D)** Go enrichment analysis for up-regulated DEGs. **(E)** Partial likelihood deviation of LASSO-Cox coefficient curves. **(F)** A red dotted line painted at the value selected for 10-fold cross-validation to select the genes for the final construction of the prognostic model. **(G)** Prognostic model construction by multifactorial Cox regression analysis.

A total of 395 DEGs were screened between high and low MSC infiltration groups by using the “DESeq2” R package (**Figures 2A, B**). To explore the relevant risk pathways and details, we performed KEGG enrichment analysis. The results showed that most upregulated genes were significantly enriched in cytokine-receptor interaction processes and other signalling pathways (**Figure 2C**). The results of GO enrichment analysis showed that most upregulated genes mainly focused on cellular component (**Figure 2D**).

### Establishment and validation of a 3-gene GBM prognostic risk model

**Figure 3:**
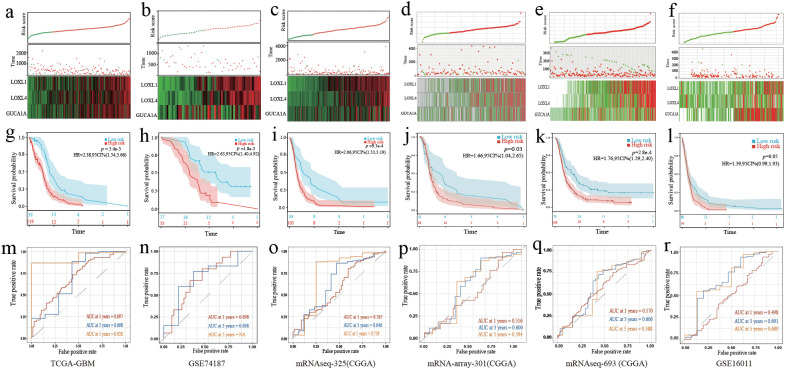
Validation of the 3-gene GBM prognostic model. **(A–F)** The distribution of the risk score, OS time, status of patients, and heatmap of the 3 risk genes expression profiles. **(G–L)** K-M curves showed survival differences in different risk groups. **(M–R)** TimeROC curves of the prognostic gene model. **(A, G, M)** Training cohort. **(B, H, N)** Validation cohort 1. **(C, I, O)** Validation cohort 2. **(D, J, P)** Validation cohort 3. **(E, K, Q)** Validation cohort 4. **(F, L, R)** Validation cohort 5.

After univariate Cox regression analysis and K-M survival analysis, we screened 38 prognostic genes from DEGs. When the lambda value was 0.207, we obtained three genes: LOXL4, LOXL1, and GUCA1A (**Figures 2E, F**). Through multivariate Cox analysis, the GBM prognostic model formula was as follows: Risk score=0.131*LOXL1+0.170*LOXL4+0.136*GUCA1A (**Figure 2G**). The risk score distribution is displayed in **Figures 3A–F**. The prognosis was worse in the high-risk group (**Figures 3G–L**). The AUC value demonstrates that this prognostic model has good risk differentiation at 1,3,5 years (**Figures 3M–R**).

### Single-cell RNA-seq analysis

**Figure 4:**
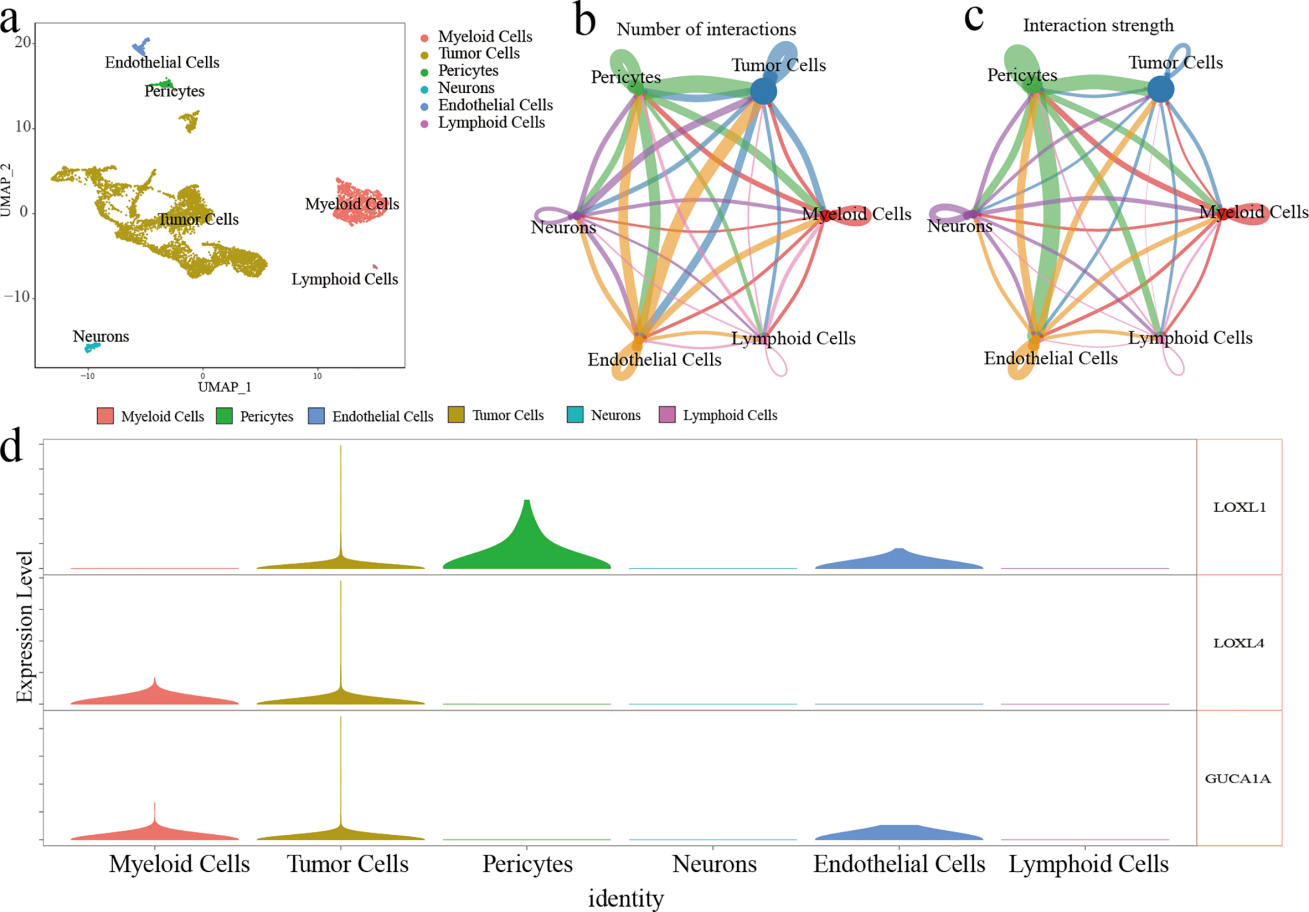
ScRNA-seq analysis of GBM. **(A)** Six cell cluster were identified on the UMAP plot. **(B, C)** The number and strength of interactions in six cell clusters **(D)** The expression level of three risk genes in six cell clusters.

Six cell clusters (myeloid cells, tumor cells, pericytes, neurons, endothelial cells, and lymphoid cells) were identified on the UMAP plot (**Figure 4A**). The number and strength of interactions in six cell clusters are shown in **Figures 4B** and **C**. The 3 risk genes have different expression levels in different cell clusters. Elevated expression of LOXL1, LOXL4 and GUCA1A was detected in tumour cells. In myeloid cells, increased expression of LOXL4 and GUCA1A was detected. In epithelial cells, elevated expression LOXL1 and GUCA1A was detected (**Figure 4D**).

### Predicting the response to immune checkpoint inhibitor therapy

**Figure 5:**
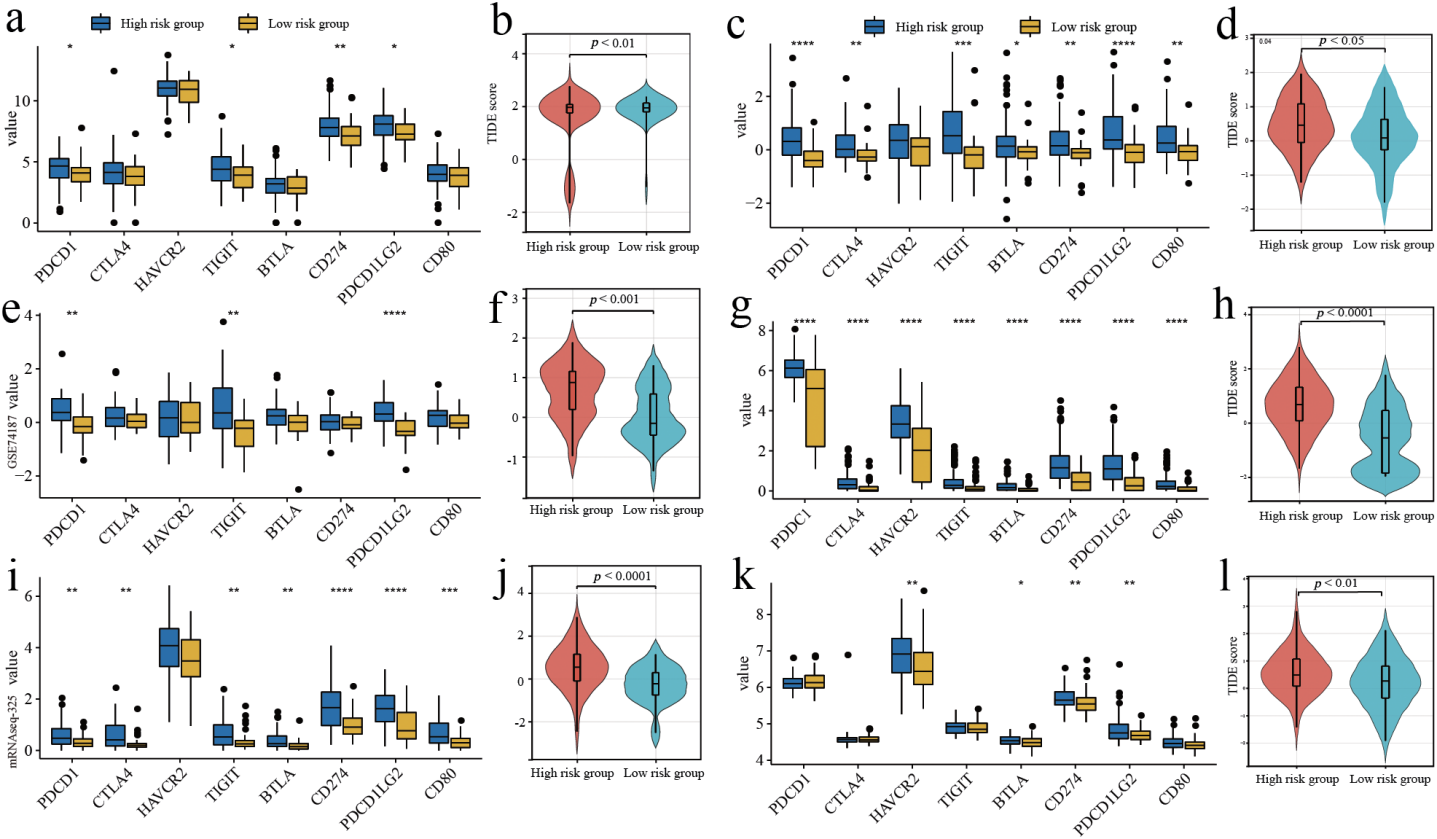
Predicting the response to immune checkpoint inhibitor therapy. **(A, C, E, G, I, K)** Comparison of the expressed amount of 8 immune checkpoint molecules. **(B, D, F, H, J, L)** Comparison of TIDE scores in different risk groups. **(A, B)** Training cohort. **(E, F)** Validation cohort 1. **(I, J)** Validation cohort 2. **(C, D)** Validation cohort 3. **(G, H)** Validation cohort 4. **(K, L)** Validation cohort 5. *p<0.05, **p<0.01, ***p<0.001 and ****p<0.0001.

Box plots were used to demonstrate the expression of immune checkpoints and related ligands. Overall, immune checkpoints and related ligand molecules were more highly expressed in the high-risk groups. PDCD1LG2, PDCD1, TIGIT, and CD274 showed more significant differences in high and low risk groups across different cohorts (**Figures 5A, C, E, G, I, K**). In all cohorts, the high-risk groups all had higher TIDE scores, which revealed that the high-risk groups were less responsive to ICI treatment (**Figures 5B, D, F, H, J, L**).

### Functional enrichment analysis of model genes and interaction genes

**Figure 6:**
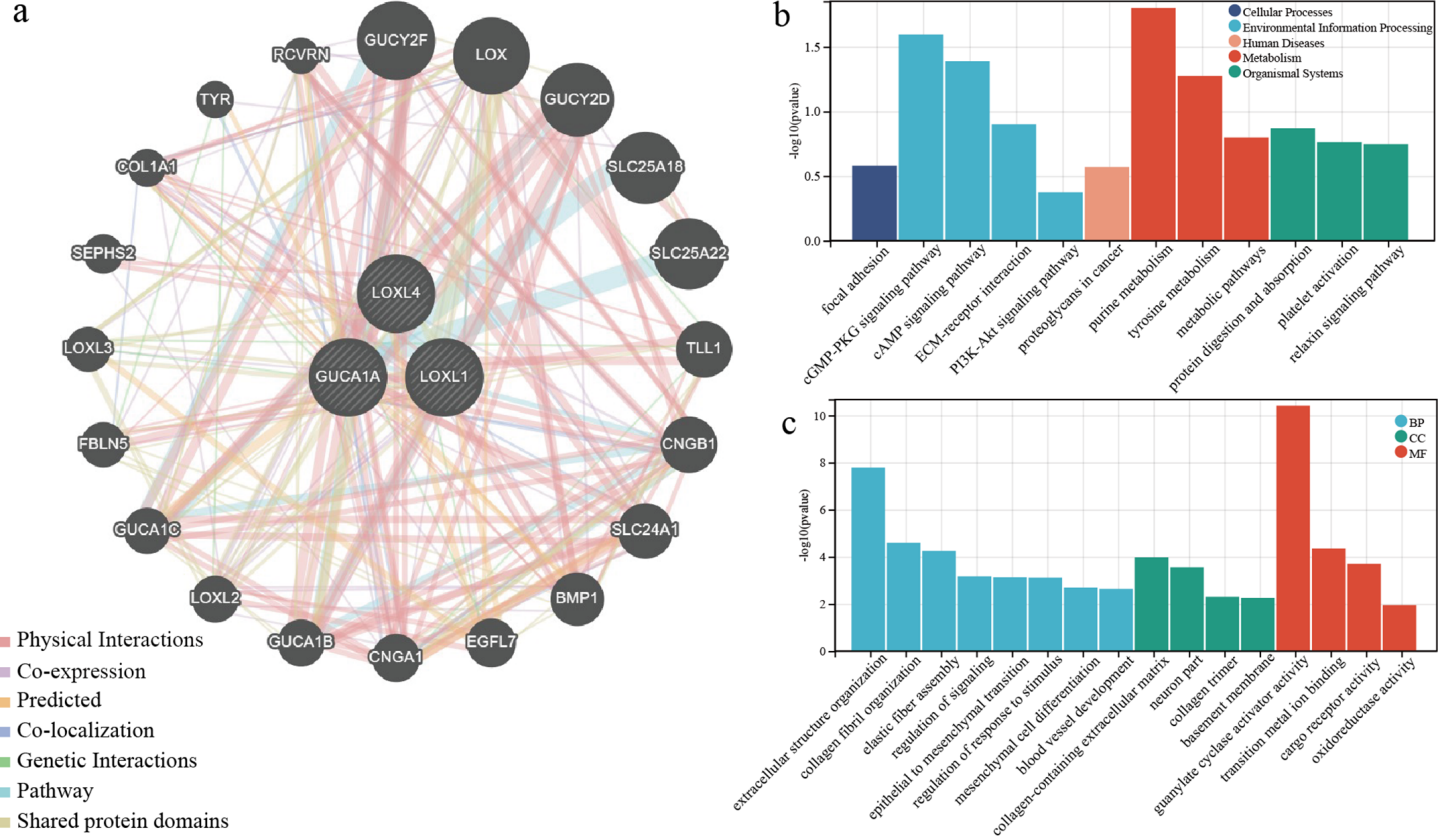
Functional enrichment analysis of model genes and interaction genes. **(A)** Prediction of interaction protein networks associated with three risk genes. **(B)** KEGG enrichment analysis for three risk genes and interaction genes. **(C)** GO enrichment analysis for three risk genes and interaction genes.

To predict the interaction genes of the 3 risk genes, we used GeneMANIA and output the visualisation results (**Figure 6A**). There are complex associations in physical interaction, co-expression and pathways, etc. The results of enrichment analysis displayed the model genes and interaction genes mainly participated in environmental information processing, metabolism and extracellular structure organisation, etc (**Figures 6B, C**).

### The expression and localisation of the 3 risk genes in GBM patients

**Figure 7:**
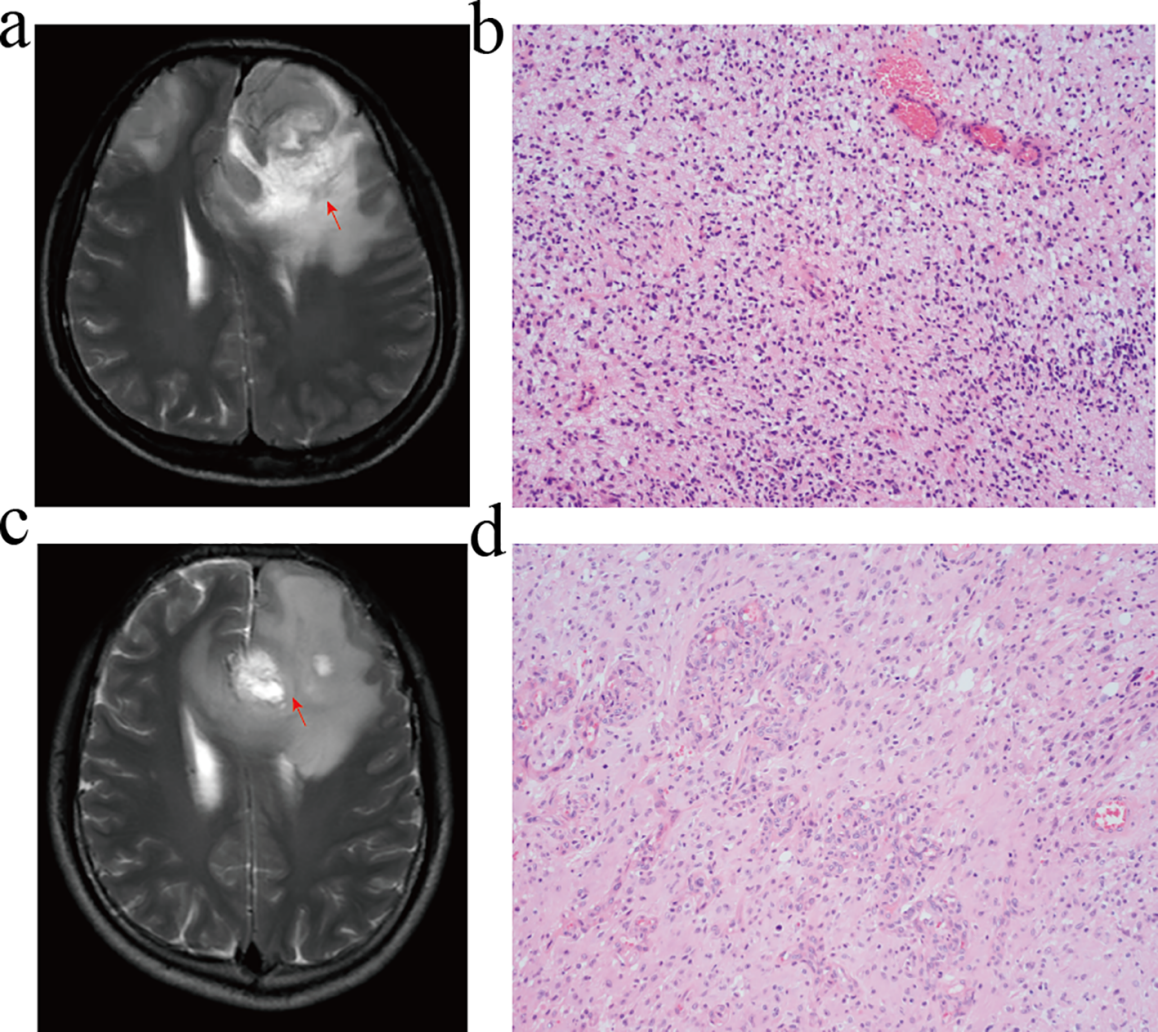
MRI and HE staining of GBM patients. **(A, B)** GBM patients with overall survival <15 months. **(C, D)** GBM patients with overall survival ≥ 15 months. **(A, C)** T2-WI; **(B, D)** HE staining, GBM cell had active nuclear division and typical nuclear morphology disappeared.

**Figure 8:**
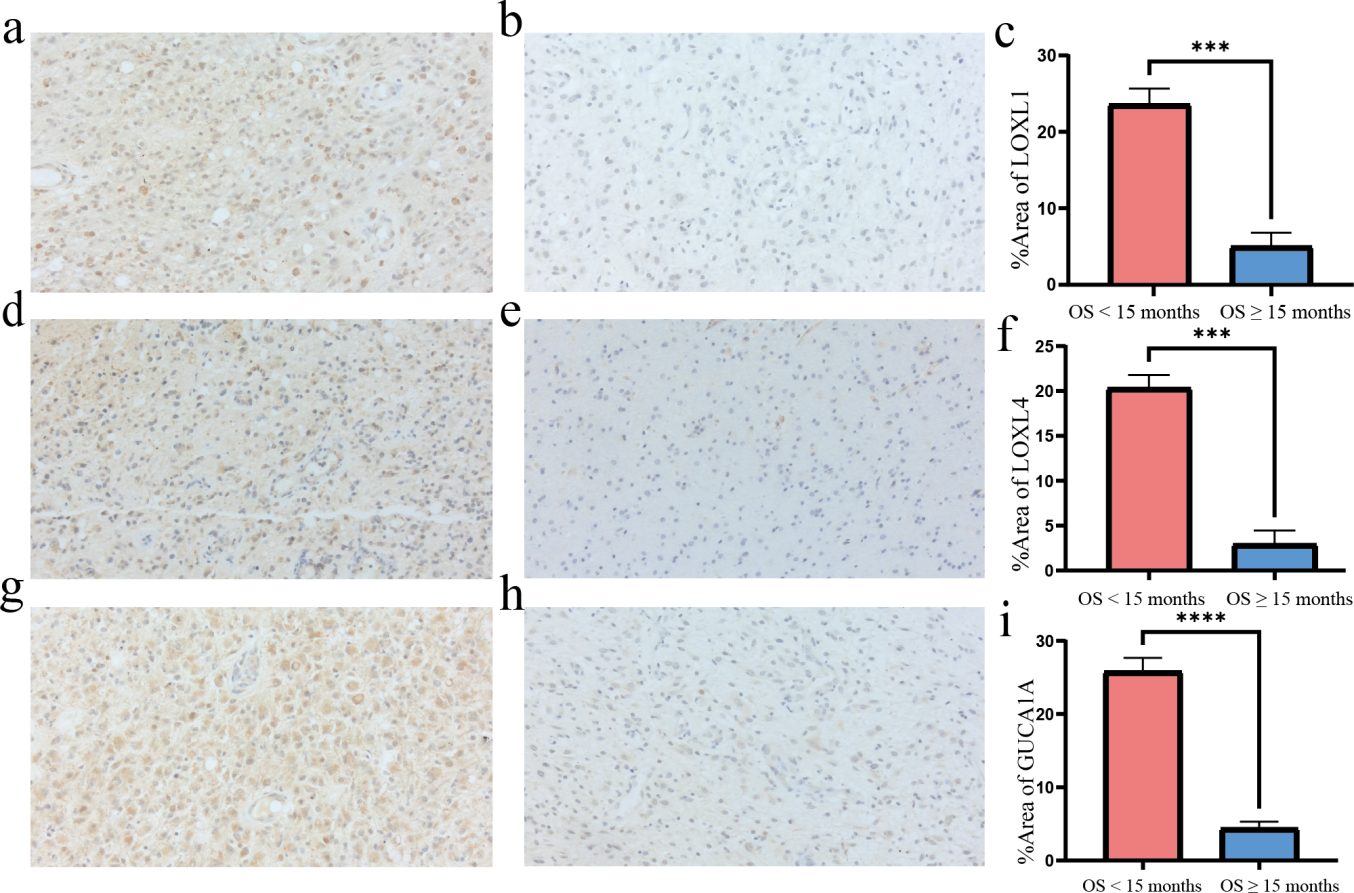
Immunohisctochemical detection of risk gene in GBM tissues. **(A, B)** LOXL1 immunohistochemistry: the positive expression was mainly in the cytoplasm and a few in the nucleus; **(C, F, I)** The %Area of risk gene in different group; **(D, E)** LOXL4 immunohistochemistry: positive localization in cytoplasm; **(G, H)** GUCA1A immunohistochemistry: positive localization in cytoplasm. Overall survival <15 months group compared to overall survival ≥15 months group ***p<0.001 and ****p<0.0001, N=3.

The GBM tumours located in the frontal lobe and temporal lobe (**Figures 7A, C**). We observed that the nuclear division of GBM cells was active. The shape of the nucleus was significantly atypical (**Figures 7B, D**). The positive localisation of LOXL1 was predominantly in the cytoplasm, with some nuclear plasma being expressed. The positive localisation of LOXL4 and GUCA1A was in the cytoplasm. Comparing the area fraction (%Area) of risk genes in GBM patients, it was observed that LOXL1, LOXL4, and GUCA1A were significantly elevated in patients with an OS <15 months (**Figure 8**).

## Discussion

The tumor-promoting role of MSCs in GBM has been widely discussed in the past decade. However, the gene expression pattern of GBM based on MSC infiltration is still unclear (2, 6, 22). Based on MSC infiltration, we screened LOXL1, LOXL4, and GUCA1A as risk biomarkers for GBM to construct a prognostic model, which was further validated by external independent cohorts and IHC. MSCs contribute to the malignant progression of GBM by promoting the formation of stromal structures favourable for tumour cell dissemination and an immunosuppressive status. Our prognostic model may contribute to the risk stratification, prognosis prediction, and screening of ICI sensitivity of patients with GBM.

We found that MSCs may be the cellular component that has a significant effect on the overall survival of GBM patients. By analysing the immune and stromal cell infiltration in the GBM environment, the results showed that the GBM was in an immunosuppressed state, but MSC infiltration was very pronounced. Hossain et al. observed that MSCs isolated from fresh human GBM tissue promoted the growth and transformation of glioma stem cells into a mesenchymal phenotype, which showed more aggressive behaviour compared to other phenotypes (3). Researchers have discovered that MSCs can be recruited around tumor cells and secrete soluble proteins for tumor progression (23, 24). GBM produces large amounts of cytokines in the TME that recruit MSCs across the blood-brain barrier to the mesenchyme of the tumor tissue to further interact with tumor cells (2, 25–28). Mechanistic studies indicate that MSCs can migrate toward the GBM vasculature and transform into pericytes to produce shorter and more blood vessels than human epithelial cells. Cytokines secreted by tumor cells can induce the conversion of MSCs into CAFs to enhance the growth and angiogenesis of tumours. The abundance of tumor blood vessels is closely related to prognosis (4, 26, 29–31).

Bulk and single-cell RNA analyses have shown that intercellular signalling plays an important role in tumor progression. For example, the phosphatidylinositol 3-kinase (PI3K)/AKT signalling pathway, which plays an essential role in regulating the survival, proliferation, and migration of tumor cells (32, 33). In addition, it has been found that deregulated ECM may contribute to the transformation of the environment into an environment that promotes cancer (34). The upregulated DEGs primarily influence extracellular structure and communication, are involved in the robustness of extracellular collagen fibril structure, and regulate the TME into an environment more suitable for tumor cell growth (35). Collagen is at low levels in the normal brain. Up-regulation of collagen gene expression has been detected in gliomas and forms a collagen-rich matrix in the tumour microenvironment, which rapid migration of tumour cells in the brain tissue (36).

We continuously narrowed down the candidate genes through a series of analyses. A prognostic model containing three genes (LOXL1, LOXL4, and GUCA1A) was finally constructed for the risk stratification of GBM patients. The lysyl oxidase-likes (LOXLs) belong to the lysyl oxidase (LOX) family and are copper-dependent monoamine oxidases that promote the cross-linking of collagen and elastin to maintain the structural stability and rigidity of the ECM (37). As glioma malignancy increased, LOX family expression increased thereby promoting ECM stiffening. The stiffened ECM can disrupt vascular integrity and lead to the formation of a hypoxic environment, enhancing GBM malignant progression (38, 39). LOXL1 accelerates the proliferation of glioma cells by modulating the Wnt/β-catenin signalling pathway. Experiments showed that LOXL1 was upregulated by the VEGFR-Src-CEBPA axis and interacted with BAG2 proteins. LOXL1 prevented BAG2-K186 ubiquitylation and promoted tumor cell survival (40). Many studies have shown that LOXL4 is overexpressed and promotes tumor progression in some human malignancies, such as hepatocellular carcinoma and gastric cancer (41, 42). Earlier studies found that exposure of macrophages to LOXL4 induced an immunosuppressive phenotype in tumours and activated the expression of programmed death ligand 1 (PD-L1), which further suppressed CD8+ T-cell function and contributed to the formation of an immunosuppressive microenvironment (43). LOXL4, which is directly regulated by TGF-β1, is involved in vascular processes associated with vascular endothelial cell remodelling and fibrosis (44).

Guanylate cyclase activator 1A (GUCA1A) regulates the neuronal calcium sensing of the phototransduction cascade (45). Previous studies showed that cone-rod dystrophy and macular dystrophy were associated with the GUCA1A gene mutation. In recent years, Liu et al. found that GUCA1A is significantly increased in osteoarthritis and is involved in the development and progression of osteoarthritis (46). Further research is needed to discover the molecular functions of GUCA1A to discover therapeutic targets for GBM. Although rare in-depth studies on the direct mechanism of model genes in GBM are currently available, our findings may provide a new perspective for exploring new relevant mechanisms for research in this field.

The enrichment analysis found that risk genes and interaction genes may be involved in various metabolic and environmental information processing, and may be associated with alterations in the structure of the tumor environment. Various metabolisms within brain tumours are reprogrammed to adapt to stress conditions, such as hypoxia, low glucose, low pH, or purine metabolism, maintaining tumor cell growth (47, 48).

In our study, the expression of multiple immune checkpoint molecules and their ligands was found to be generally increased in the high-risk group, with PDCD1, TIGIT, CD274, and PDCD1LG2 being significantly increased in at least five data cohorts. Combined with TIDE analysis, patients in the high-risk group were predicted to be less sensitive to ICI therapy. We therefore considered that the risk genes may be involved in regulating the process of aberrant activation of immune checkpoint molecules, which promotes the evasion of tumour cells from the surveillance of the immune system (49). Immune checkpoints are a class of immunosuppressive molecules that are expressed on immune cells to keep the level of immune system activation within the normal range and avoid overactivation of the immune system. However, in many malignant tumours, tumour cells are able to regulate the overexpression of immune checkpoints, blocking the process of antigen presentation to T cells, reducing T cell reactivity, and causing the tumour microenvironment to become highly immunosuppressive, which facilitates tumour cell survival by escaping from the surveillance of the immune system (49, 50). GBM may increase the risk of immune evasion through the regulation of risk genes and immune checkpoint molecules, leading to tumor progression (7). However, ICI has not yet achieved significant efficacy in the therapeutic application of GBM, which may be related to the existence of the blood-brain barrier, the low degree of T-cell infiltration and the complex highly immunosuppressive environment (51). However, this does not indicate that ICI therapy is completely ineffective in GBM, and a significant increase in overall survival was observed in GBM mouse models with the combination of anti-VEGF and ICI (52). In the future, the prognostic model constructed in this study will be useful for the selection of ICI treatment options and provide individualised treatment recommendations for GBM patients.

Our study is helpful for understanding the immune environment of GBM patients. MSCs have a great impact on the progression and prognosis of GBM. Here, we constructed a three-risk gene model for risk stratification and ICI application guidance in GBM patients. However, there is also the drawback of not being able to distinguish the origin of MSCs during immune infiltration assessment. Our work may provide new targets for the treatment of GBM. This is the first time that LOXL1, LOXL4 and GUCA1A have been explored as key risk genes for deteriorating the prognosis of GBM at the level of infiltration differences in MSCs. Risk genes may accelerate tumour cell growth and infiltration by promoting the formation of environmental structures more conducive to tumour cell spread and the construction of an immunosuppressive state. More in-depth research is needed to transform this conclusion into clinical practice. By assessing the expression levels of risk genes in GBM patients, it may be possible to predict the responsiveness of GBM patients to ICI therapy and provide risk stratification management and clinical treatment guidance.

## Data Availability

Publicly available datasets were analyzed in this study. This data can be found here: TCGA-GBM, http://cancergenome.nih.gov/. GSE74187, https://www.ncbi.nlm.nih.gov/geo/query/acc.cgi. mRNAseq-325, mRNA-array-301, mRNAseq-693, http://www.cgga.org.cn/download.jsp. GSE16011, https://www.ncbi.nlm.nih.gov/geo/query/acc.cgi.
